# Potential risk of residual cancer cells in the surgical treatment of initially unresectable pancreatic carcinoma after chemoradiotherapy

**DOI:** 10.1186/s12957-015-0617-3

**Published:** 2015-06-26

**Authors:** Hironobu Takano, Takahiro Tsuchikawa, Toru Nakamura, Keisuke Okamura, Toshiaki Shichinohe, Satoshi Hirano

**Affiliations:** Department of Gastroenterological Surgery II, Hokkaido University Graduate School of Medicine, N-15 W-7, Sapporo, Hokkaido 060-8638 Japan

**Keywords:** Initially unresectable pancreatic carcinoma, Chemoradiotherapy, Adjuvant surgery

## Abstract

**Background:**

With development of chemoradiotherapy for pancreatic carcinoma, borderline resectable or initially unresectable cases sometimes become operable after long-term intensive chemoradiotherapy. However, there is no established strategy for adjuvant surgery with respect to whether the surgical resection should be extensive or downsized accordingly with diminished disease areas following response to chemoradiotherapy.

**Methods:**

The clinical and pathological aspects of 18 patients with initially unresectable pancreatic cancer who underwent adjuvant surgery after chemo(radio)therapy in our department from 2007 were evaluated.

**Results:**

Overall survival from initial treatment was much better for patients with R0 resection than for patients with R1/2 resection. In two of three patients who had complete improvement of plexus (PL) invasion after chemo(radio)therapy, there had still remained pathological plexus invasion. It was shown that tumors did not shrink continuously from the tumor front, but parts remained discontinuously at the distal portion in the process of tumor regression by chemo(radio)therapy.

**Conclusions:**

In adjuvant surgery for patients with locally advanced pancreatic cancer, the potential risk of residual cancer in the regression area following chemoradiotherapy should be considered. Achieving R0 resection will lead to an improved prognosis, and it is necessary to consider how well the extent of resection is after a favorable response to chemoradiotherapy.

## Background

Pancreatic carcinoma is a disease with a very poor prognosis. Although surgery is the only curative option for this disease, only 20 % of patients can be treated surgically [1]. The prognosis of patients with unresectable disease is significantly worse than that of patients with resectable disease. In the past, various treatments for unresectable pancreatic carcinoma had been adopted, but prognosis in patients with unresectable pancreatic carcinoma continues to be disappointing [2, 3]. Recently, with the development of chemoradiotherapy for pancreatic carcinoma, “borderline resectable” or “initially unresectable” cases sometimes become operable after long-term, intensive chemoradiotherapy [4, 5]. We have previously reported the outcomes of adjuvant surgery for initially unresectable pancreatic carcinoma with long-term chemoradiotherapy [6]. We defined “adjuvant surgery” as radical surgery for initially unresectable cases of pancreatic carcinoma which had a well response to chemo(radio)therapy without original intent to proceed to resection. There are several reports showing a correlation between prognosis and neural invasion or histopathological grading after neoadjuvant chemoradiotherapy of patients with pancreatic carcinoma [7, 8]. However, there is no established strategy for “adjuvant surgery” with respect to whether the surgery should be extensive or whether the surgical resection could be downsized to correspond to the diminished disease areas following response to chemoradiotherapy.

Our surgical strategy for initially unresectable patients with a long-term favorable response to chemo(radio)therapy included the resection area initially affected by the tumor, because it was unknown whether cancer still remained or not in the area where suspected involved lesion had improved on imaging with good response to chemo(radio)therapy. In this report, the suitability of our surgical strategy for initially unresectable pancreatic carcinoma following chemoradiotherapy was evaluated based on the pathological analysis of the resected specimens.

## Methods

### Patients

From July 2007 to April 2013, surgery was performed following chemo(radio)therapy for 18 patients with initially unresectable pancreatic carcinoma in our department. Surgical indications for resectable tumors were determined based on the following criteria: no distant metastasis, no extension to the common hepatic artery (CHA) or the superior mesenteric artery (SMA) (head of the pancreas), and no extension to the gastro-duodenal artery (GDA) or the SMA (body and tail of the pancreas). For borderline resectable tumors, the surgical indications were based on the following criteria: no distant metastasis and extension to half around plexus invasion of the SMA. Other tumors were deemed unresectable. Patients with unresectable tumors subsequently underwent chemo(radio)therapy. Resectability of the tumors was determined by imaging and intraoperative findings and then radical surgery was performed, in principle, six or more months after the start of the initial treatment. Imaging diagnosis was done by a radiologist, surgeon, gastroenterologist, and an echo technician at a Cancer Board conference. In this study, all 18 patients were initially diagnosed unresectable tumors through this process. At the adjuvant surgery, our operative procedure included the resection area initially affected by the tumor. This study was approved by the Institutional Ethics Committee of Hokkaido University Hospital.

### Assessment of clinical and pathological efficacies after chemoradiotherapy

Radiological assessment after initial treatment was performed according to Response Evaluation Criteria In Solid Tumors (RECIST) [9]. The pathological effect of preoperative therapy was assessed by Evans grading system [10]. These assessments were done by a radiologist, surgeon, gastroenterologist, pathologist, and an echo technician at a Cancer Board conference and at a clinicopathological conference, respectively.

### Survival analysis

Overall patient survival was calculated from the date of initial treatment to the date of last follow-up (censored) or the date of patient death (event). Differences in survival times between patient subgroups were analyzed using the log-rank test. Survival probabilities were calculated using the Kaplan-Meier method. In all tests, statistical significance was set at *P* < 0.05. All analyses were performed using JMP® 10 software.

## Results

### Patients’ characteristics

Patients’ data and the perioperative data are shown in Tables 1 and 2. The median age of the 18 patients was 63 years (range: 43–68 years). The numbers of males and females were nearly equal. The tumor location was the head of the pancreas in 7 patients and the pancreatic body or tail in 11 patients. Histological tumor types of all patients before starting treatment were invasive ductal adenocarcinoma. Tumors were initially unresectable due to distant metastasis (hepatic metastasis or para-aortic lymph node metastasis) in 5 patients, para-aortic lymph node metastasis in 2, hepatic metastasis in 3, and major artery involvement in 13 patients. Of them, celiac artery invasion in 1, GDA invasion in 4, CHA invasion in 4, SMA invasion in 3, and portal vein (PV) tumor thrombus in 1 patient, respectively. We regarded these 13 patients who had invasion to major vessels and had no distant metastasis as the locally advanced cases. All patients were initially given chemo(radio)therapy. The treatment regimens are shown in Table 3. All 18 patients underwent chemotherapy including gemcitabine (GEM); 4 patients were treated with chemoradiotherapy. The median period for preoperative therapy was 9 months (range: 6–44 months).

**Table 1:** Patient characteristics

Characteristics		2007–2013 *n* = 18
Age (years)		63 (43–68)
Sex	Male	8
	Female	10
Tumor location	Ph	7
	Pb	8
	Pt	3
Reason for initially unresectable	Locally advanced	13
	metastatic	5
Preoperative therapy	Chemo (IV)	11
	Chemo (TA)	3
	Chemoradi	4
RECIST	SD	5
	PR	13
	CR	0

*Ph* pancreas head, *Pb* pancreas body, *Pt* pancreas tail, *SD* stable disease, *PR* partial response, *CR* complete response

**Table 2:** Perioperative data

Postoperative date		2007–2013 *n* = 18
Operative procedure	SSPPD	7
	DP-CAR	7
	DP	3
	TP-CAR	1
Operation time (min)		418 (200–879)
Blood loss (ml)		905 (330–3200)
Complication	Total	11 (61 %)
	Pancreatic fistula	4 (22 %)
	SSI	4 (22 %)
	Ischemic gastritis	1 (5 %)
Hospital stay (days)		30 (12–97)
Mortality		0
Evans grade	I	4
	IIa	6
	IIb	5
	III	1
	IV	2
Residual tumor	R0	15
	R1	2
	R2	1

*SSPPD* subtotal stomach-preserving pancreaticoduodenectomy, *DP-CAR* distal pancreatectomy with en bloc celiac axis resection, *DP* distal pancreatectomy, *TP-CAR* total pancreatectomy with en bloc celiac axis resection

**Table 3:** Neoadjuvant therapy

Chemo(radio)therapy	Regimen	*n*	Period of therapy (month)
Chemotherapy (IV)	GEM + S-1	10	8.5 (5–32)
	GEM	1	11
Chemotherapy (TA)	GEM + 5-FU	3	7/34/44
Chemoradiotherapy	GEM + S-1	2	7/16
	GEM + 5-FU	1	8
	GEM	1	32
			9 (5–44)

*GEM* gemcitabine, *5-FU* 5-fluorouracil

### Surgical procedures performed

Subtotal stomach-preserving pancreaticoduodenectomy (SSPPD) was performed for 7 patients, distal pancreatectomy with en bloc celiac axis resection (DP-CAR) was performed for 7 patients, total pancreatectomy with en bloc celiac axis resection (TP-CAR) was performed for 1 patient, and distal pancreatectomy (DP) was performed for 3 patients. SSPPD was performed using the modified Child method. Concomitant vascular resection was basically performed if main vascular invasion or plexus invasion was suspected, except for the SMA. The SMA nerve plexus was removed circumferentially from the root of the SMA longitudinally along to the branch of the inferior pancreaticoduodenal artery in DP-CAR. Among the 18 patients, there were patients with main vessel resection and reconstruction including PV resection and reconstruction for 12 patients and arterial resection and reconstruction for 4 patients.

Among the three patients with hepatic metastasis, two patients initially had solitary metastasis in segment III and segment IV, respectively. The other patient had hepatic metastasis both in segment I and segment VIII, resulted in no pathological evidence of malignancy followed by concomitant partial hepatic resection. Previous two patients with solitary hepatic metastasis underwent resection for primary lesion alone, because the hepatic lesion was not identified in CT or completely shrunken before surgery.

### Objective tumor response to chemoradiotherapy

R0 resection was achieved in 15 patients (83 %), while R1 resection was achieved in 2 patients (12 %) and R2 resection was achieved in 1 patient (5 %). The reasons for R1 resection were a pathologically positive diagnosis at the PV stump and a pathologically positive diagnosis at the arterial plexus around the SMA, respectively. And the reason for R2 resection was peritoneal metastasis identified just before abdominal closure.

Using Evans grading system for tumor response, 4 patients had grade I, 6 patients had grade IIa, 5 patients had grade IIb, 1 patient had grade III, and 2 patients had grade IV. Based on RECIST, 13 patients had partial response (PR), and 5 patients had stable disease (SD). No patients had complete response (CR), but 2 patients had pathological CR.

### Improvement of arterial plexus invasion on CT imaging over time

**Fig. 1:**
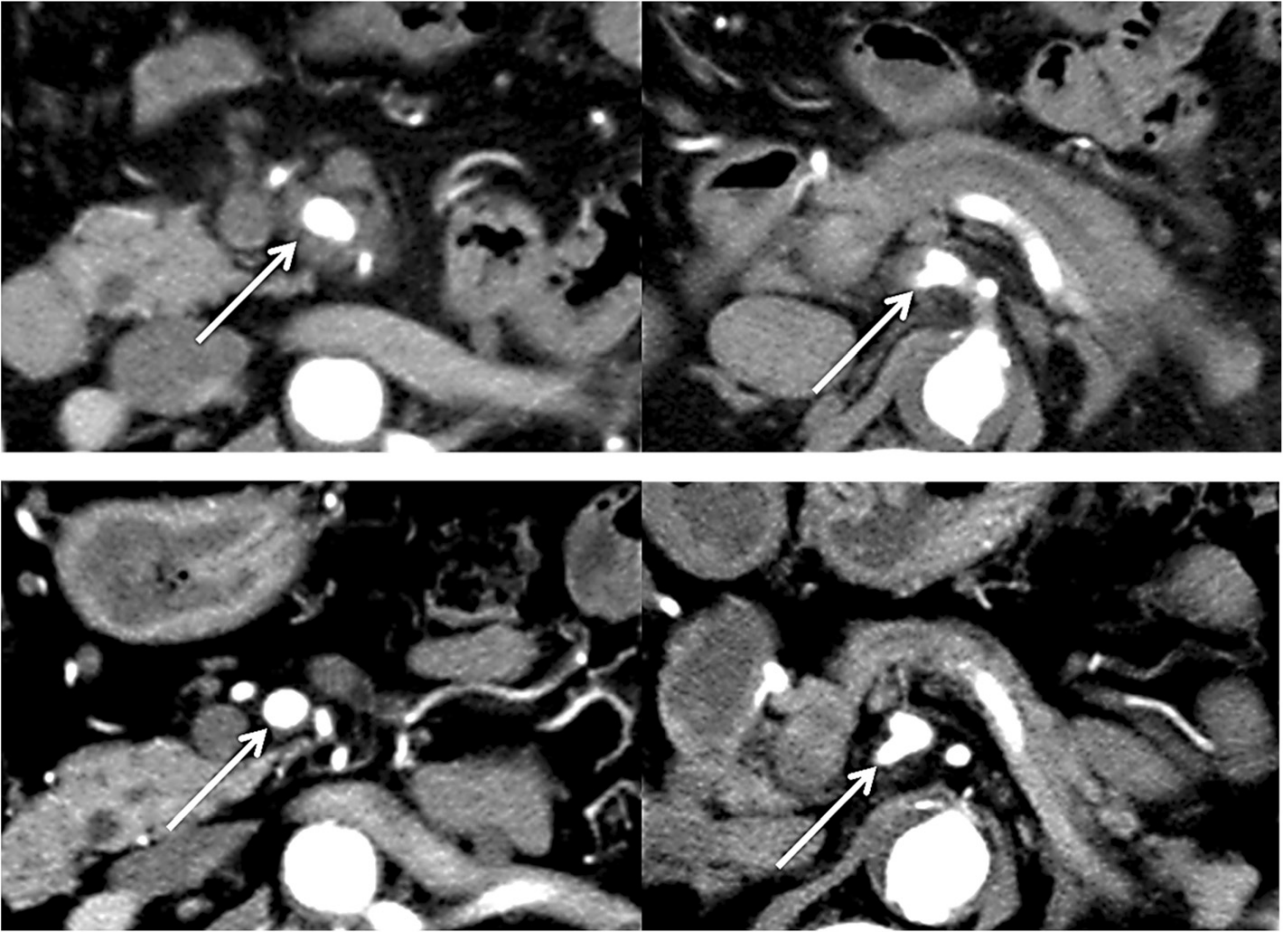
CT scan shows tumor invading to the SMA and CA (*above*). CT scan shows the tumor decreased by chemotherapy (*below*). White arrows indicate tumor invasion to the SMA or CA

**Fig. 2:**
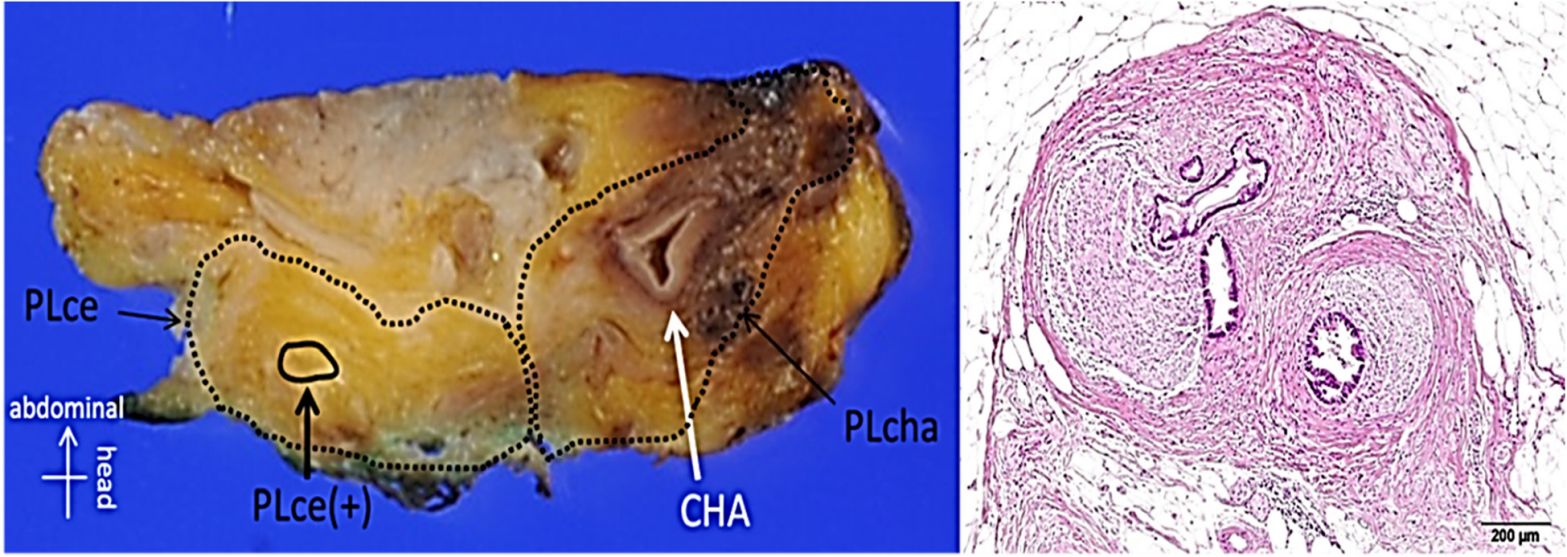
Histopathological mapping on the macroscopic section at the plexus around the CA, indicating residual cancer cells at the plexus around the CA (left, area encircled with *solid line*). hematoxylin-eosin staining showed the residual cancer cells present at the area encircled with the *broken line* (right)

Of the 18 patients, 12 patients (66 %) were initially diagnosed as having unresectable disease with arterial plexus invasion around the CA in 1 patient, the GDA in 4 patients, the SMA in 3 patients with pancreatic body cancer, and the CHA in 4 patients with head of the pancreas cancer. After the chemoradiotherapy, these findings on CT imaging had improved with the plexus around the artery of all patients. These fields, initially considered to include cancer cells, were totally resected in an en bloc-wise manner using already described procedures. Among them, 2 out of 3 patients in complete improvement after chemoradiotherapy and 7 out of 9 patients in partial improvement had still remained pathological plexus invasion (Table 4). Here, we show one characteristic case (no. 10 in Table 4) that was initially diagnosed as having unresectable disease with invasion to the plexus around the SMA. After chemotherapy including GEM and TS-1 for 15 months, adjuvant surgery was performed because PR was achieved (Fig. 1). CT scan after chemotherapy showed tumor shrinkage, which was amenable to radical resection by DP. However, DP-CAR was performed because of the policy described above. The pathological findings after resection showed tumor cells remaining around the celiac artery discontinuously (Fig. 2).

**Table 4:** Data of patients with initially unresectable due to arterial plexus invasion

No.	Tumor location	Suspected arterial plexus invasion	Operative procedure	Combined resection	Improvement of arterial plexus invasion on CT imaging	Invasion to the extra pancreatic nerve in pathological diagnosis	Residual tumor	Outcome from IT (month)
1	Pb	Ce	DP-CAR	Ce	Partial	PLce (+)	R1	44 DRD
2	Pb	GDA	DP-CAR	Ce	Partial	PLce, cha (+)	R0	54 AFD
3	Pb	GDA	DP-CAR	Ce, PV	Partial	PL (−)	R0	92 AFD
4	Pb	GDA	DP-CAR	Ce, PV	Partial	PLcha, sma (+)	R0	55 AFD
5	Pb	GDA	TP-CAR	Ce, GDA, PV	Partial	PLce, sma (+)	R0	67 AFD
6	Pb	SMA	DP-CAR	Ce, PV	Partial	PLspa (+)	R0	91 AFD
7	Ph	CHA	SSPPD	PV	Completely	PL (−)	R0	63 DRD
8	Ph	CHA, PHA	SSPPD	CHA, PHA, RHA, PV	Partial	PL (−)	R0	31 ARD
9	Ph	CHA	SSPPD	CHA, PV	Partial	PLcha (+)	R0	18 DRD
10	Pb	SMA	DP-CAR	Ce, PV	Completely	PLce (+)	R0	32 AFD
11	Pb	SMA	DP-CAR	Ce, PV	Completely	PLce (+)	R0	15 ARD
12	Ph	CHA	SSPPD	CHA, LHA, MHA, PV	Partial	PLcha (+)	R0	11 AFD

*Ce* ceriac artery, *GDA* gastro-duodenal artery, *CHA* common hepatic artery, *PHA* proper hepatic artery, *RHA* right hepatic artery, *LHA* left hepatic artery, *MHA* middle hepatic artery, *PV* portal vein, *AFD* alive free of disease, *ARD* alive with recurrent disease, *DRD* dead of recurrent disease

### Clinical factors associated with overall survival

**Fig. 3:**
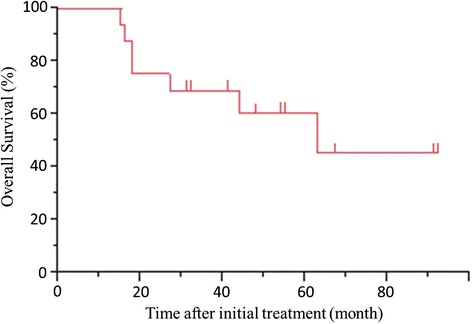
Overall survival from initial treatment of all patients. The 5-year survival rate is 60.3 %. The median follow-up time from initial treatment is 39 months (range: 11–92 months)

**Fig. 4:**
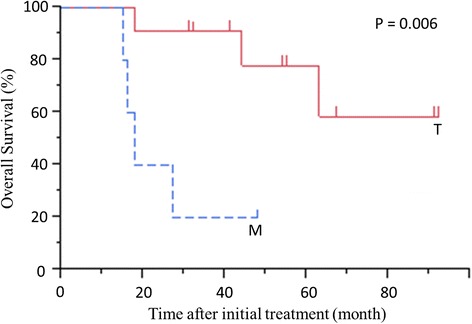
Overall survival beginning at initial treatment in patients with locally advanced disease (T) or metastatic disease (M) as the reason for initial unresectability. *P* = 0.006

Overall survival of all patients is shown in Fig. 3. The median follow-up time from initial treatment in all 18 patients was 39 months, and the 5-year survival rate was 60.3 %. The survival from initial treatment was significantly worse for patients with metastatic disease than for patients with locally advanced disease (*P* = 0.006) (Fig. 4).

**Fig. 5:**
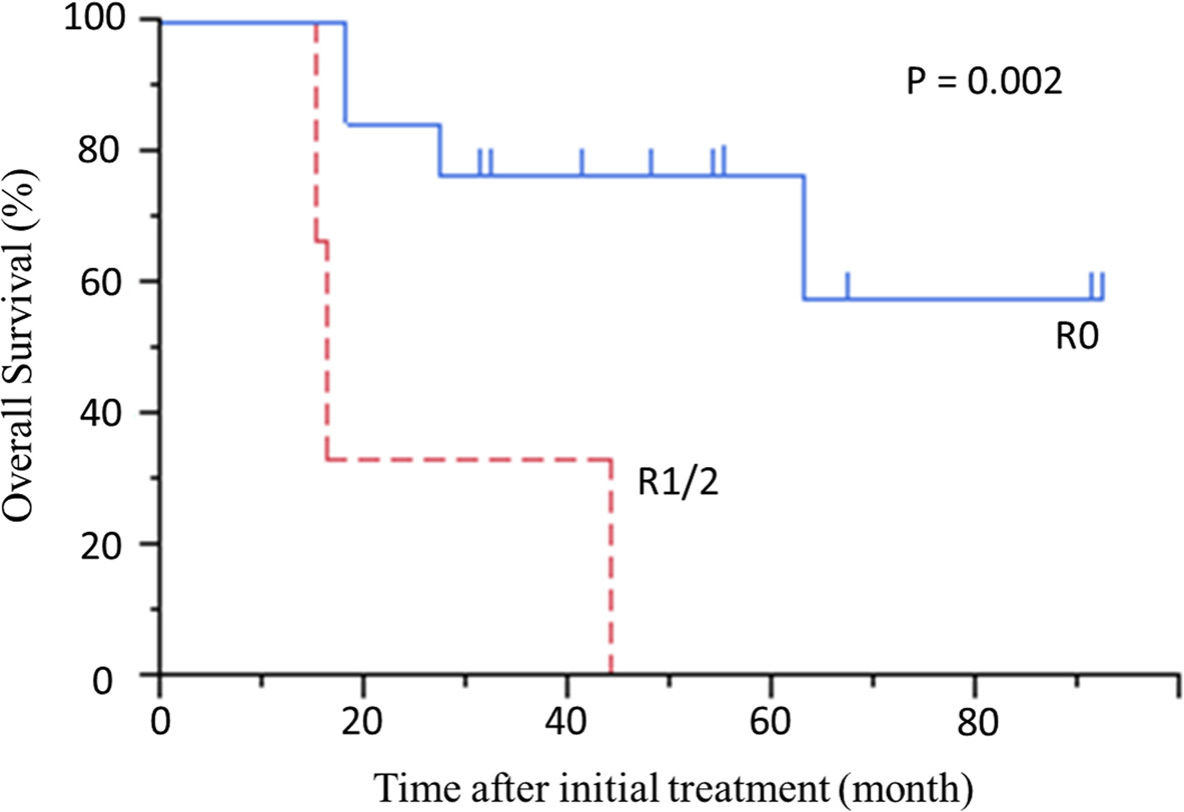
Overall survival beginning at initial treatment in patients with R0 resection or R1/2 resection. *P* = 0.002

Survival from initial treatment was much better for patients with R0 resection than for patients with R1/2 resection (*P* = 0.002) (Fig. 5).

**Fig. 6:**
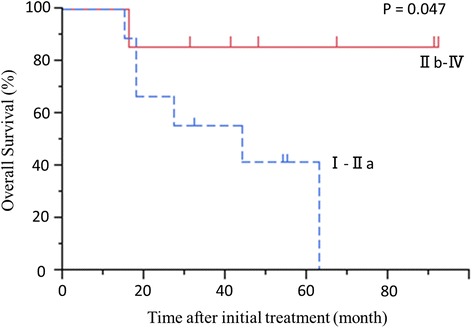
Overall survival beginning at initial treatment in patients with Evans grade I-IIa or grade IIb-IV. *P* = 0.047

Examining the pathology, there was a significant difference in the prognosis between Evans grade I-IIa and grade IIb-IV (*P* = 0.046) (Fig. 6). However, there was no significant difference in the prognosis between patients with and without plexus invasion (*P* = 0.750).

## Discussion

The prognosis of patients with unresectable pancreatic cancer is extremely poor, and development of new anti-cancer drugs including molecular target inhibitors has been in progress. Among clinical trials worldwide [11–17], several anti-cancer agents have been reported to have better response rates and 2-year survival rates with chemotherapy alone (41.9–81.3 % and 23.5–27.1 %, respectively) [18, 19]. However, there have been few cases with 5-year survival treated by chemoradiotherapy alone. Therefore, we have performed adjuvant surgery for selected cases with a long-term favorable response to chemoradiotherapy since 2006. Satoi et al. reported, in their Project01 study by the Japanese Society of Hepato-Biliary-Pancreatic Surgery, that the overall survival rate from the initial treatment with adjuvant surgery was higher than that with non-surgical anti-cancer treatment in patients who received chemoradiation therapy for more than 8 months [20]. Deyali reported in their series of 20 patients who were surgically resected after neoadjuvant chemotherapy that there was a correlation between patient prognosis and Evans grade between Evans grade I-IIb and grade III-IV [8]. Although the preoperative factors that could predict patient prognosis were not identified, it is of great interest to predict the timing of the surgical intervention by preoperative biomarkers, because it is not clear whether the operation is truly good for patients with a good response to chemotherapy. A further prospective study is warranted in the future.

In the present series of 18 cases, the 5-year survival rate from initial treatment was 60.3 %, and there were 2 cases who survived more than 5 years after the operation. Surgical margin-negative was a significant predictor of a good prognosis. The period of preoperative treatment of these patients other than the R0 operation was 5–11 months, but it is unclear whether R0 resection is possible if the preoperative treatment period is increased. Considering patients with R0 resection, the R0 rate of patients with distant metastasis was 60 % (3/5) and that with locally advanced disease was 92 % (12/13). Therefore, adjuvant surgery might be appropriate for patients with locally advanced pancreatic cancer, and it is important to achieve R0 resection. For hepatic metastasis, only one patient underwent concomitant hepatic resection with no pathological finding of malignancy. Other two patients did not undergo hepatic resection because of no findings of hepatic metastasis in CT before surgery. In terms of postoperative disease prognosis for the three patients with initial hepatic metastasis, one patient who underwent hepatic resection with no pathological evidences for malignancy died of para-aortic lymph node recurrence in 21 months after surgery. Other two patients without hepatic tumors after chemoradiotherapy developed multiple liver metastases including initial site in 4 months or 2 months, respectively, after primary site resection. The fact showed that they might have partial response for hepatic metastasis which was too small to detect in CT but not always showed complete response.

This was a retrospective analysis, so it is not clear whether a similar prognosis could be obtained if chemotherapy was continued in the patients with adjuvant surgery. Further study is needed to decide whether surgery should be performed after chemoradiotherapy in every case.

Regarding the surgical strategy following chemotherapy for initially unresectable pancreatic cancer, the extent of resection remains controversial. Whether the shrunken or arrested area which might have well responded to the chemoradiotherapy should also be included in the scheduled resection area is of the greatest interest. Although these kinds of surgical strategies concerning resection area after neoadjuvant chemoradiotherapy are well established in other cancers [21, 22], it is still controversial for initially unresectable pancreatic cancer. For pancreatic cancer, there have been only several reports of a small number of patients and limited cases with surgical procedures. Furthermore, there are few reports that suggest an appropriate treatment strategy, including the extent of resection for the initially unresectable cases after chemoradiotherapy.

In our department, we resect the area initially affected by tumor and regional major vessels even if tumor shrinkage was obtained and perineural invasion around the artery on imaging modality was improved by initial treatment. As shown in one characteristic case that had undergone DP-CAR with dissection of the initially suspected area with perivascular neural invasion, clear evidence was shown for the first time that the tumor did not shrink linearly or continuously from the tumor front but that part of it often remained discontinuously at the distal portion in the process of tumor regression by chemotherapy. It is unknown how remaining tumor cells in this way can affect recurrence or a patient’s prognosis. Because of the good prognosis of patients with R0 resection, it has become clear from the pathological aspects that the operative procedure including fields initially affected by tumor should be concomitantly resected as much as possible, even if arterial resection and reconstruction are indicated.

There are some reports on the pros and cons of additional arterial resection, but satisfactory results have not been obtained for SMA resection, with a high rate of surgery-related mortality [23, 24]. Therefore, in our department, we perform additional arterial resection actively only for non-SMA cases. In the present study, 4 of the 18 patients underwent arterial resection and reconstruction, and R0 resection was achieved for all 4 patients. However, accumulation of further cases is needed to consider the long-term results for patients with arterial resection.

The limitations of this study were, firstly, the small number of the cases, secondly, selection vias with which we indicated surgery for patients who had favorable response to chemo(radio)therapy, and lastly, the retrospective nature of the study design. Therefore, statistical analysis in this study might be better interpreted as a trend instead of a statistical significance. And this study is the retrospective study for patients with unresectable pancreatic carcinoma. Future prospective study should be performed to clarify whether to continue chemotherapy or indicate radical R0 operation for patients with good response to chemo(radio)therapy.

## Conclusions

In conclusion, in adjuvant surgery for patients with locally advanced pancreatic cancer, achieving R0 resection will improve the prognosis. Because of the potent residual cancer cells at the initial tumor front and the limitations of imaging diagnosis, it is necessary to consider how well the extent of resection is after a favorable response to chemo(radio)therapy.
